# Colloidal Structure Dictates Antimicrobial Efficacy in LL‐37 Self‐Assemblies With Glycerol Monooleate

**DOI:** 10.1002/smll.202405131

**Published:** 2024-10-15

**Authors:** Jules D. P. Valentin, Parth Kadakia, Lucie J. Varidel, Marc C. A. Stuart, Stefan Salentinig

**Affiliations:** ^1^ Department of Chemistry and National Center of Competence in Research Bio‐inspired Materials University of Fribourg Chemin du Musée 9 Fribourg 1700 Switzerland; ^2^ Centre for System Chemistry Stratingh Institute for Chemistry and Groningen Biomolecular Science and Biotechnology Institute University of Groningen Nijenborgh 7 Groningen 9747AG The Netherlands

**Keywords:** antimicrobial nanomaterials, antimicrobial peptides, biofilm, glycerol monooleate, LL‐37, SAXS, structure‐function relationship

## Abstract

The antimicrobial peptide LL‐37 is a promising alternative to conventional antibiotics to combat bacteria in suspension and biofilms. Its self‐assembly with polar lipids is suggested to improve its potential for therapeutic applications with higher stability against degradation and bioavailability. This study investigates the self‐assembly of LL‐37 with glyceryl monooleate (GMO), establishing the link between colloidal structure and antimicrobial activity. Small‐angle X‐ray scattering, dynamic light scattering and cryogenic transmission electron microscopy show structural transformation from dispersions of inverse bicontinuous structure (cubosomes) to multilamellar vesicles and direct rod‐like mixed‐micelles upon increasing the content of LL‐37 in GMO. *In vitro* assays against planktonic and biofilm cells demonstrate that 128 µg mL^−1^ of GMO cubosomes have no impact on *Pseudomonas aeruginosa*. Still, the cubosomes reduce the *Staphylococcus aureus* planktonic population by ≈ 1‐log after 24 h. Cylindrical micelles formed at LL‐37/GMO 9/1 and 8/2 with 128 µg mL^−1^ LL‐37 decrease the *Pseudomonas aeruginosa* population by 6‐log. This activity is gradually abolished when LL‐37 is encapsulated in vesicles or cubosomes. They also demonstrate low antibiofilm efficacy and promote the biomass of *Staphylococcus aureus* biofilms. These results highlight the importance of colloidal structure for therapeutic outcomes, providing insights for advanced lipid nanocarrier designs.

## Graphical abstract

This study investigates the structure‐function relationship in LL‐37/glycerol monooleate self‐assemblies, aiming to optimize the antimicrobial efficacy of the self‐assembled materials. Upon increasing the LL‐37 content relative to glycerol monooleate, transitions from multilamellar vesicles to cylindrical micelles are observed. These structural transformations correlate with increasing antimicrobial activity. This demonstrates that the antimicrobial peptide‐to‐lipid ratio and subsequent self‐assembled structures are crucial for therapeutic outcomes.

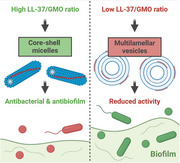

## 1 Introduction

Antimicrobial resistance (AMR) is one of the leading public health threats of the 21^st^ century.^[^ ^1^, ^2^, ^3^ ^]^ New antibiotics are urgently needed to combat AMR infections, but their development remains challenging due to scientific, economic, and regulatory obstacles.^[^ ^4^ ^]^ In this context, antimicrobial peptides (AMPs) have emerged as promising alternatives to conventional antibiotics to combat bacterial infections.^[^ ^5^, ^6^ ^]^ Most notably, LL‐37 is a well‐studied cationic AMP from the cathelicidin family, showing broad‐spectrum bactericidal activity and immunomodulatory functions.^[^ ^7^, ^8^ ^]^ Its mechanism of action involves disrupting bacterial membranes, interacting with intracellular targets, and interfering with the cell signaling system.^[^ ^9^, ^10^, ^11^ ^]^ These multiple targets make the emergence of bacterial resistance to LL‐37 less likely compared to conventional antibiotics.^[^ ^7^ ^]^ Moreover, LL‐37 shows strong activity against biofilms, bacterial communities embedded in a self‐produced matrix highly resistant to antimicrobials and responsible for most persistent infections.^[^ ^12^, ^13^, ^14^ ^]^ Despite these promising characteristics, LL‐37's clinical use is limited by its stability and bioavailability, and the development of nanocarriers to address these challenges is an active field of research.^[^ ^15^, ^16^, ^17^ ^]^

Lipid‐based nanostructures formed through the self‐assembly of biocompatible polar lipids, such as glycerol monooleate (GMO), that self‐assemble into lyotropic liquid crystalline structures in excess water have been researched as drug nanocarriers.^[^ ^18^, ^19^, ^20^ ^]^ In contrast to polymeric nanocarriers, lipid‐based nanocarriers have the unique property of fusing with the bacterial membrane to deliver the encapsulated drugs directly into the bacteria.^[^ ^21^, ^22^, ^23^ ^]^ GMO is one of the most studied amphiphilic lipids for pharmaceutical formulations.^[^ ^24^ ^]^ It is non‐toxic, biocompatible, biodegradable, and approved for topical and oral drug products by the U.S. Food and Drug Administration (FDA).^[^ ^25^ ^]^ The interactions between LL‐37 and GMO have been widely investigated and shown potential for clinical application.^[^ ^26^, ^27^, ^28^, ^29^, ^30^ ^]^ LL‐37 was found to spontaneously integrate into the GMO‐based cubosomes, modifying their self‐assembled structure in a composition‐dependent manner.^[^ ^26^, ^31^, ^32^ ^]^ The resulting antimicrobial nanomaterials were demonstrated compatible with human dermal fibroblasts, even promoting cell proliferation.^[^ ^28^ ^]^ Encapsulation of LL‐37 within these cubosomes has been shown to protect the peptide from enzymatic degradation.^[^ ^33^, ^34^ ^]^ The drug delivery mechanism of LL‐37 likely originates from the adsorption and fusion of the nanoparticles with the bacterial membranes, underscored by the low leakage of LL‐37 from GMO‐based nanocomplexes.^[^ ^26^, ^28^, ^32^, ^35^ ^]^

This study investigates the structure‐function relationship in LL‐37/GMO self‐assemblies at various compositions, aiming to optimize antimicrobial efficacy against bacteria in suspension and in biofilms. The physicochemical characteristics of the nanocarriers and their impact on bacterial physiology are investigated. The particle charge, size, and structure are measured via ζ‐potential, dynamic light scattering (DLS), cryogenic transmission electron microscopy (cryo‐TEM), and small‐angle X‐ray scattering (SAXS). Furthermore, the link between these properties and antimicrobial activity against *Pseudomonas aeruginosa* and *Staphylococcus aureus*, two leading pathogens in AMR‐related deaths, is investigated for planktonic bacteria and biofilms.

## 2 Results

### 2.1 Impact of the LL‐37/GMO Ratios on the Self‐Assembly

**Figure 1:**
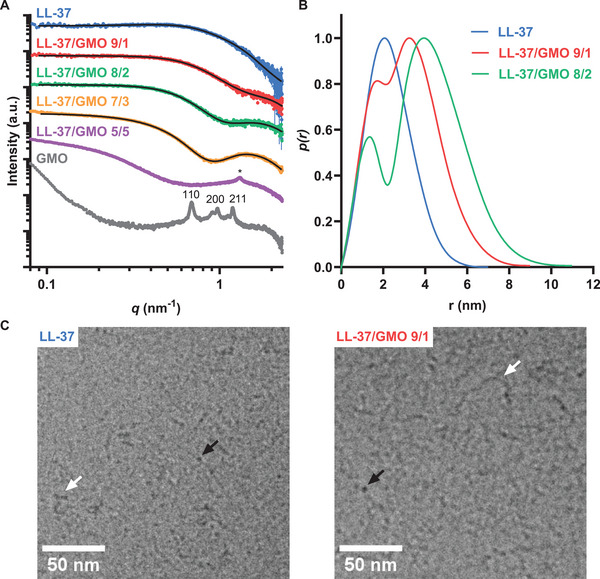
Composition‐dependent self‐assembly of LL‐37/GMO systems. A) Experimental SAXS curves (scatters) with the calculated fits from GIFT analysis using a hard‐sphere S(q) model (black line). The resulting S(q) parameters are presented in Table S1 (Supporting Information) with the S(q) shown in Figure S2 (Supporting Information). B) The corresponding *p(r)* functions (normalized to the maximum of the main peak) for LL‐37/GMO 10/0, 9/1, and 8/2. C) Representative cryo‐TEM images of LL‐37 and LL‐37/GMO 9/1 self‐assemblies. Black and white arrows highlight small micelles of ≈5 nm and long worm‐like micelles of ≈50 nm, respectively. Micrographs of all self‐assemblies can be found in Figure S4 (Supporting Information). All samples were in PBS in the presence of F127 at pH 7.0.

The composition‐dependent self‐assembly of LL‐37/GMO dispersions stabilized with the commonly used triblock copolymer F127 in phosphate‐buffered saline (PBS) solution at pH 7.0 was determined using SAXS, DLS, and cryo‐TEM. Consistent with a previous report,^[^ ^28^ ^]^ the SAXS curve of the F127 stabilized GMO dispersions showed the presence of three Bragg peaks indicative of the 110, 200, and 211 *hkl* reflections of the inverse bicontinuous cubic *Im3m‐type* structure with a lattice parameter of 13.0 ± 0.1 nm (**Figure** **1**A). The SAXS curves of LL‐37 in PBS, both with and without F127, are shown in Figure 1A and Figure S1 (Supporting Information), respectively. F127 was included also in the LL‐37 suspensions to further analyze its effect in the following microbiological studies. This was also for the ease of preparation of the L‐L37/GMO structures, and to reduce the number of variables upon comparing the activity of the different self‐assembled structures. The SAXS data were then analyzed with the generalized indirect Fourier transformation (GIFT) method. As the concentrations of the self‐assembled structures were above ≈3 wt%, a structure factor, S(q), model was included in the calculations to account for inter‐particle correlations. The resulting pair distance distribution function, *p(r)*, is the real space representation of the form factor scattering, allowing a direct determination of the size and shape of the scattering objects.^[^ ^36^ ^]^ The form factor scattering, after the removal of the structure factor, was further analyzed with Guinier analysis.

The experimental SAXS data and the best possible fit for LL‐37 dispersions with and without F127, measured directly after preparation, are shown in Figure 1A and Figure S1 (Supporting Information), respectively. The shape of the *p(r)* presented in Figure 1B and Figure S1 (Supporting Information) is characteristic of cylindrical micelles^[^ ^30^ ^]^ with a cylinder length of ≈7 nm (from *p(r)* = 0) for both LL‐37 dispersions with and without 10 wt% F127 relative to LL‐37. The resulting structure factor parameters are shown in Table S1 (Supporting Information) with the S(q) plots in Figure S2 (Supporting Information). Additionally, the form factor scattering was approximated with an analytical model for a homogenous cylinder, Equation 6.^[^ ^37^ ^]^ The best possible fit of this model to the experimental SAXS data shown in Figure S3 and Table S2 (Supporting Information) summarizes the resulting fitting parameters. A cylinder length of 5.6 ± 0.02 nm and 5.3 ± 0.01 nm for LL‐37 with and without F127, respectively, was produced, which is in reasonable agreement with the findings from the *p(r)* analysis. A radius r = 1.5 ± 0.1 nm and 1.6 ± 0.1 nm was exhibited by both LL‐37 dispersions with and without F127, respectively, which agrees with a previous report showing r = 1.3 ± 0.2 nm.^[^ ^30^ ^]^ Additional cryo‐TEM analysis of the LL‐37 samples with and without F127 confirms the observation of worm‐like micelles (Figure 1C and Figure S4, Supporting Information). They appear with a diameter of ≈3 nm but polydisperse in length. Some of them appear several tens of nanometers long. These cryo‐TEM images were taken five days after preparation, and the cylindrical micelles may grow over time which has been reported for LL‐37 before.^[^ ^38^ ^]^ The results demonstrate that adding 10% of F127 relative to LL‐37 content had little impact on the size and shape of the cylindrical LL‐37 micelles.

The LL‐37/GMO self‐assemblies that formed upon mixing the GMO cubosomes and LL‐37 were also analyzed with SAXS (see Figure 1 and **Table** **1** for composition). The SAXS curves for the systems at LL‐37/GMO 9/1, 8/2, and 7/3 ratios show a broad correlation peak between *q* = 0.9 and 2.4 nm^−1^. The observed features suggest the presence of micelles with inhomogeneous excess electron density in the cross‐section region, similar to some LL‐37/OA micelles reported in our previous study.^[^ ^22^ ^]^ The GMO alkyl chains together with hydrophobic amino acids of LL‐37 may form the core of the micelles with lower relative electron density compared to PBS, and the headgroups with counter ions with higher relative electron density. From GIFT analysis, the shape of the *p(r)*, calculated for 9/1, 8/2, and 7/3 ratios, supports the presence of core–shell type micelles (Figure 1; Figure S5A, Supporting Information). A local minimum in the *p(r)* at r ≈ 2 nm occurred and was more pronounced at the higher GMO concentration (Figure 1B; Figure S5A, Supporting Information). The tailing in the *p(r)* at r > 4 nm, r > 5 nm, and r > 7 nm for LL‐37/GMO 9/1, 8/2, and 7/3 ratios, respectively indicates an elongated structure with a maximum dimension at *p(r)* = 0 of ≈9, 11 and 16 nm for LL‐37/GMO 9/1, 8/2, and 7/3, respectively (see Figure 1; Figure S5, Supporting Information). For the sample at LL‐37/GMO 7/3 this upturn of the I(q) at low q‐values (< 0.1 nm^−1^) is visible in Figure 1. This results from larger structures with dimensions above the resolution limit of our SAXS set‐up. Hence the p(r) was mathematically truncated for this sample and does not reflect the maximum dimension but still demonstrates the worm‐like shape (see Figure S5, Supporting Information).

**Table 1:** Composition of the studied systems.

LL‐37/GMO preparations	LL‐37 [mg mL^−1^]	GMO [mg mL^−1^]	F127 [mg mL^−1^]	Total concentration [wt.%]
Unformulated LL‐37	30.0	0	0	3.00
LL‐37+F127	30.0	0	3.00	3.30
LL‐37/GMO 9/1	30.0	3.34	3.00	3.63
LL‐37/GMO 8/2	30.0	7.50	3.00	4.05
LL‐37/GMO 7/3	30.0	12.9	3.00	4.59
LL‐37/GMO 5/5	30.0	30.0	3.00	6.30
GMO+F127	0	30.0	3.00	3.30

The cylindrical cross‐section pair‐distance distribution function, *p_c_* (*r*), was calculated to gain further insights into the morphology of the LL‐37/GMO 9/1 self‐assemblies. The *p_c_* (*r*) was then deconvoluted to obtain the radial excess electron density distribution along the cylinder cross‐section, Δρ_*c*_(*r_c_*) (Figure S6, Supporting Information). A cylinder diameter of 7 nm was estimated from *D*_max_ at *p_c_* (*r*) =  0 for LL‐37/GMO 9/1 ratio (Figure S6A, Supporting Information). Moreover, a cylinder radius of ≈3.1 nm with a core radius of ≈1.5 nm and a corresponding shell thickness of≈1.6 nm was determined for LL‐37/GMO 9/1 (Figure S6B, Supporting Information). This radius is in reasonable agreement with the $\frac{D_{max}}{2}=3.5$ from the *p_c_* (*r*).

Additional SAXS analysis of the LL‐37/GMO 9/1 system in the absence of F127 was performed to reveal the impact of the block co‐polymer on the structure (Figure S7, Supporting Information). A local minimum in the *p(r)* at r ≈ 2 nm was observed with a tailing at r > 4.0 nm and a maximum dimension at p(r) = 0 of 6 nm, comparable to the sample with F127 presented above. Cryo‐TEM images of the LL‐37/GMO 9/1 sample, five days after preparation, in the presence and absence of F127, displayed worm‐like micelles with a large distribution in length, some even above 100 nm, and a diameter of 5 nm (Figure 1C; Figure S4, Supporting Information). While the diameter of the micelles agrees with the results from SAXS analysis, they appear much longer in the cryo‐TEM analysis. To further clarify this, additional SAXS data were recorded three weeks after preparation for this sample. These data show an upturn at low q values, reflecting particles that are larger than the maximum dimension that is approachable with this set‐up (Figure S8, Supporting Information). This shows that the LL‐37/GMO 9/1 worm‐like micelles grow in length over time.

The form factor scattering data of the micelles, after removal of the structure factor contribution, were further analyzed with Guinier analysis after preparation. The micelle's radius of gyration (R_g_), the cylinder cross‐section (R_gc_), and the dimension of the largest semiaxis (c) of the elongated structures were calculated. The R_g_ was ≈1.8 nm for both LL‐37 with and without F127, respectively, suggesting minimal structural change due to the addition of F127 (Table S3 and Figure S9, Supporting Information). A systematic increase in R_g_ was observed at various LL‐37/GMO mixtures. The R_g_ values increased from 2.4 nm at LL‐37/GMO 9/1 to 4.7 nm at LL‐37/GMO 7/3 ratio (Table S3 and Figure S9, Supporting Information), in agreement with the trend from p(r) analysis above. From the cross‐section Guinier plot [ln P(q)q] vs q^2^, the R_gc_ values also showed an increasing trend ranging from 0.6 to 3.2 nm between LL‐37 and LL‐37/GMO 7/3 ratio (**Table** **2**). The cross‐section Guinier plots and corresponding fits are shown in Figure S10 (Supporting Information). Moreover, the dimension of the largest semiaxis of the elongated cylinders, calculated with Equation 12, increased from 3.9 (LL‐37 to 7.7 nm, further supporting the increase in elongation and dimensions of the cylindrical structures (Table 2).

**Table 2:** Particle size and ζ‐potential values of LL‐37/GMO self‐assemblies in PBS at pH 7.0 (composition is presented in Table 1) were measured directly after preparation. The radius of gyration of the cylinder cross‐section (*R_gc_*), and the dimensions of the largest semiaxis were measured using SAXS and Guinier analysis. The hydrodynamic radius (R_H_) was measured by DLS at an angle of 90 °. D_max_ is estimated from the p(r) analysis of the SAXS data. The ζ‐potential values represent the mean ± SD of three measurements.

LL‐37/GMO preparations	R_gc_ [nm]	Largest semiaxis [nm]	D_max_ [nm]	R_H_ [nm]/PDI	ζ‐potential [mV]
Unformulated LL‐37	0.6	3.9	7	multimodal	–
LL‐37+F127	0.6	3.8	7	multimodal	11.0 ± 0.3
LL‐37/GMO 9/1	0.9	5.0	9	multimodal	16.9 ± 1.2
LL‐37/GMO 8/2	1.6	5.9	11	multimodal	16.4 ± 1.3
LL‐37/GMO 7/3	3.2	7.7	–	multimodal	22.7 ± 0.2
LL‐37/GMO 5/5	–	–	–	28 / 0.6	18.4 ± 0.7
GMO+F127	–	–	–	83 / 0.4	−4.0 ± 0.4

Upon increasing the GMO content to LL‐37/GMO 5/5, the upturn in the low *q* region (*q* < 0.15 nm^−1^) appeared directly after preparation. A Bragg reflection (indexed with *) around *q* = 1.3 nm^−1^ is seen in the SAXS data with higher‐order reflections at q = 2.6 and 3.9 nm^−1^ (Figure 1A; Figure S11, Supporting Information). The calculated characteristic spacing of $d=\frac{2\pi }{q}=$ 4.8 nm is comparable to *d* spacing of ≈6 nm for LL‐37/GMO 4/6 reported in a previous study.^[^ ^26^ ^]^ These features suggest the presence of multilamellar structures, likely in the form of oligo‐lamellar vesicles. This observation is consistent with cryo‐TEM analysis of this sample, showing vesicles and bilayer fragments of various sizes and shapes coexisting with small nanoparticles (Figure S4, Supporting Information). This is also in line with previous studies discussing the coexistence of small micelles and larger lamellar structures at LL‐37/GMO ≈ 5/5.^[^ ^26^, ^28^ ^]^ Cubosomes eventually form at even higher GMO concentrations, exceeding the mass fraction of LL‐37, which was already demonstrated in previous studies.^[^ ^26^, ^31^ ^]^

The results show that the positively charged amphiphilic α‐helical peptide LL‐37 accommodates at the lipid‐water interface of the cubosomes and tunes the interfacial curvature to be more positive, resulting in vesicles and cylindrical micelles upon increasing its concentration relative to GMO.

To further analyze the size of the structures, the hydrodynamic radius was measured by multi‐angle DLS at scattering angles between 39 ° and 124 °. The DLS intensity autocorrelation functions in Figure S12 (Supporting Information) show a single exponential decay for the GMO cubosomes and the LL‐37/GMO 5/5, indicating a mostly monomodal particle size distribution. Multiple decays reflecting multimodal particle size distributions are observed for LL‐37/GMO 7/3 and 9/1 samples. A linear fit of the average decay constant $\left(\bar{\Gamma }\right)$, calculated from these functions against *q* ^2^ was used to calculate the average hydrodynamic radius (R_H_), as shown in Figure S13 (Supporting Information). The plot demonstrates a mostly linear trend in the experimental data for GMO and LL‐37/GMO 5/5. The GMO dispersions have an R_H_ of 83 nm (PDI = 0.4). Upon increasing the LL‐37 content to LL‐37/GMO 5/5, the R_H_ decreased to 28 nm (PDI = 0.6). On the contrary, the LL‐37/GMO 7/3 system deviates from the linear relationship, owing to the multi‐modal particle size distribution. This finding agrees with the coexistence of micelles and larger particles, likely vesicles, from the SAXS analysis for this sample. However, upon cumulant analysis of the first decay, a R_H_ of 17 nm was obtained for the LL‐37/GMO 7/3 ratio at an angle of 90 °. The autocorrelation functions of the LL‐37/GMO 9/1 samples with and without F127 support the SAXS findings of worm‐like micelles. They reflect broad particle size distributions as the length of worm‐like micelles is mostly entropy controlled and thus rather polydisperse.

To gain further insights into the self‐assembly of the uncharged GMO with cationic LL‐37, the ζ‐potential of the nanoparticles was measured in PBS at pH 7.0 (Table 2). The GMO cubosomes exhibited a ζ‐potential of −4 ± 0.4 mV, in agreement with previous studies.^[^ ^39^ ^]^ LL‐37 showed a positive ζ‐potential of +11 ± 0.3 mV, and all the LL‐37/GMO ratios (9/1, 8/2, 7/3, 5/5) exhibited a positive ζ‐potential ranging between 15 and 23 mV. As the net ζ‐potential does not vary significantly despite increasing the LL‐37 concentration relative to GMO up to the 9/1 ratio, the results suggest that the number of LL‐37 molecules per particle remains relatively constant upon modification of the structure. This is in agreement with the decrease in particle size upon increasing the LL‐37 content observed with SAXS and DLS, showing small cylindrical micelles at high LL‐37 content, growing and transforming to larger vesicles at lower LL‐37 content.

Enzymatic degradation of LL‐37 in the self‐assemblies was used to study whether LL‐37 is inside the structures or on the surface of the micelles and prone to enzymatic degradation. The self‐assemblies were prepared at LL‐37/GMO 10/0, 9/1, and 5/5 ratios with a fixed LL‐37 concentration of 512 µg mL^−1^ and were treated with 20 µg mL^−1^ of proteinase K for 30 min at 25 °C. The antimicrobial activity of all systems was then determined by quantifying the number of viable planktonic cells of *Escherichia coli* after 24 h treatment at 37 °C by colony‐forming units (CFU) counting. The data revealed that the enzymatic degradation of LL‐37 was comparable in its free form and in the LL‐37/GMO 9/1 micelles. Only the LL‐37/GMO 5/5 showed no significant modification of the antimicrobial activity (Figure S14, Supporting Information). This suggests that LL‐37 is surface‐adsorbed in the micelles, thereby preserving its ability to interact with bacterial membranes but also prone to degradation.

### 2.2 Influence of Cubosomes on Bacteria and Biofilm Physiology

**Figure 2:**
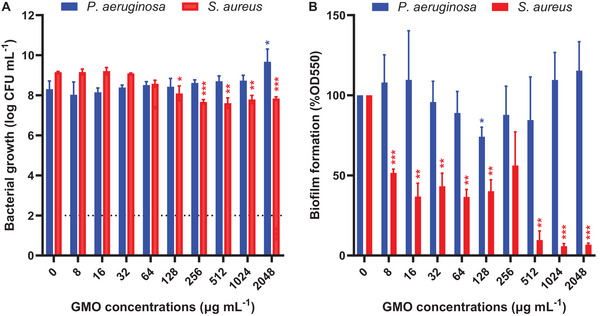
A) Bacterial growth and B) biofilm formation of *P. aeruginosa* and *S. aureus* after 24 h culture in the presence of increasing concentrations of F127‐stabilized GMO‐based cubosomes. (A) Bacterial growth is measured by counting the CFU mL^−1^ after 24 h culture in MHB media supplemented with the cubosomes. (B) Biofilm formation is measured by CV staining after 24 h culture in M9 or 10% TSB + 0.1% Glucose supplemented with the cubosomes for *P. aeruginosa* and *S. aureus*, respectively. The results were normalized to the biomass of the control set as 100%. The results represent the mean ± SD of three independent biological repeats. Student *t*‐tests were performed using the growth and biomass of the control as references with ^*^ *P* < 0.05, ^**^ *P* < 0.01, ^***^ *P* < 0.001.

The effect of GMO‐based cubosomes, stabilized with F127 in the absence of LL‐37, on *P. aeruginosa* and *S. aureus* physiology was investigated to understand further the impact of composition and structure on the antimicrobial activity. The cubosomes had little impact on *P. aeruginosa* planktonic cell growth, except at very high concentrations for which growth was promoted (1‐log increase in CFU count with 2048 µg mL^−1^ of GMO) (**Figure** **2**A). The cubosomes exhibited a minor concentration‐dependent impact on *P. aeruginosa* biofilm formation, studied with the crystal violet (CV) method. 128 µg mL^−1^ of GMO led to a 25% decrease in biomass, while 2048 µg mL^−1^ led to a 15% increase compared to the control (Figure 2B). These results suggest that GMO has a slightly beneficial role in *P. aeruginosa* physiology and alters the biofilm matrix structure.

In contrast to *P. aeruginosa*, GMO cubosomes had a significant impact on *S. aureus* planktonic growth. After 24 h culture, 128 µg mL^−1^ of GMO as cubosomes reduced the *S. aureus* planktonic population by ≈1‐log, with no further reduction with up to 2048 µg mL^−1^ (Figure 2A). Furthermore, 512 µg mL^−1^ of GMO reduced *S. aureus* biofilm formation by more than 90% after 24 h culture (Figure 2B). Immersing established biofilms in the GMO cubosome suspensions for 30 min showed increased CV staining values for *S. aureus*, but not for *P. aeruginosa* (Figure S15, Supporting Information). This suggests a fast interaction of GMO with *S. aureus* biofilms likely participating in biofilm matrix structure.

### 2.3 Impact of Self‐Assembled LL‐37/GMO Self‐Assemblies Against Planktonic Cells

**Figure 3:**
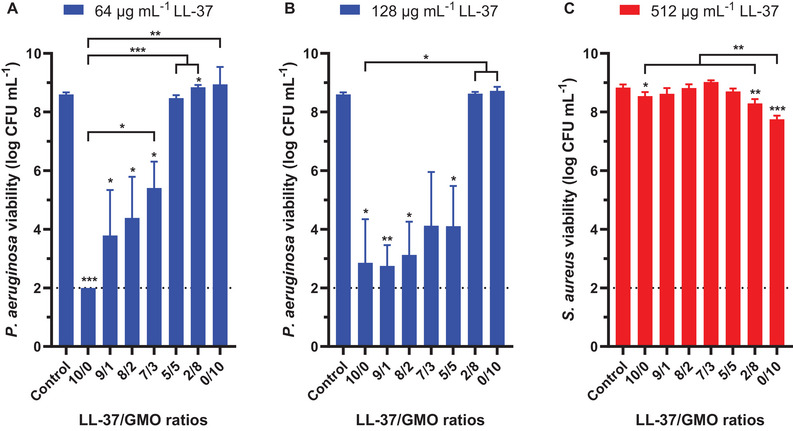
Antibacterial impact of LL‐37/GMO self‐assemblies against A,B) *P. aeruginosa* and C) *S. aureus* planktonic populations. Bacterial viability is measured by counting the CFU mL^−1^ after 24 h culture in MHB media supplemented with various LL‐37/GMO ratios with concentrations of LL‐37 fixed at (A) 64, (B) 128, and (C) 512 µg mL^−1^ and stabilized with 10% wt F127 relative to LL‐37. “0/10” represents GMO at equivalent concentrations to LL‐37, (A) 64, (B) 128, and (C) 512 µg mL^−1^ GMO stabilized with 10% wt F127. The results represent the mean ± standard deviations (SD) of three independent biological repeats. The dashed line represents the limit of detection of the assay. Student *t*‐tests were performed using the viability of the control as reference or between two conditions with ^*^ *P* < 0.05, ^**^ *P* < 0.01, and ^***^ *P* < 0.001.

The antimicrobial activity of the LL‐37/GMO self‐assemblies was investigated against two laboratory strains *P. aeruginosa* MPAO1 and *S. aureus* ATCC 35556.^[^ ^40^, ^41^, ^42^ ^]^ Increasing the GMO content in self‐assemblies prepared with a constant concentration of LL‐37 decreased the killing activity against *P. aeruginosa* (**Figure** **3**A,B). After 24 h treatment, 64 µg mL^−1^ of unformulated LL‐37 resulted in a > 6‐log reduction of the *P. aeruginosa* planktonic population compared to the control. The presence of 10 wt% F127 relative to the LL‐37 concentration (6.4 µg mL^−1^) did not alter the antimicrobial activity of LL‐37 against *P. aeruginosa* (Figure S16, Supporting Information). In contrast, 64 µg mL^−1^ of LL‐37 associated with GMO in LL‐37/GMO 9/1, 8/2, and 7/3 ratios led to 5, 4, and 3‐log reduction of *P. aeruginosa* population, respectively. Moreover, the vesicles obtained with LL‐37/GMO 5/5 and 2/8 ratios abolished the antimicrobial activity of 64 µg mL^−1^ of LL‐37 (Figure 3A). The antibacterial impact of LL‐37/GMO 5/5 nanocomplexes was restored using a higher overall concentration of the nanocarriers, at 128 µg mL^−1^ of LL‐37, exhibiting similar reduction as the 10/0, 9/1, 8/2, and 7/3 ratios (Figure 3B). This is consistent with a previously published work, in which cubosomes at an LL‐37/GMO 5/95 ratio exhibited no killing activity against *E. coli*, while the 5/5 and 10/0 systems were active.^[^ ^26^ ^]^ The reduced antimicrobial efficacy with increasing GMO concentration in the LL‐37/GMO assembly cannot be solely attributed to GMO or F127 alone, as individually testing these components at equivalent concentrations did not impact *P. aeruginosa* (Figure 3; Figure S17, Supporting Information). It is rather a correlation between the changes in colloidal structure from micelles to vesicles and their effect on biological activity. To further analyze the effect of elongation of the worm‐like micelles over time of storage, additional antibacterial assays were performed comparing formulations directly after preparation and after one week of storage at 4 °C (Figure S18, Supporting Information). The activity of all analyzed formulations against *P. aeruginosa* was lost after one week of storage.

On the other hand, LL‐37 was demonstrated to have no bactericidal activity against *S. aureus* with up to 512 µg mL^−1^ (Figure 3C). This observation was similar for LL‐37 alone and mixed with F127 (Figure S16, Supporting Information). These results contrast with previously published results showing the antimicrobial activity of down to 8 µg mL^−1^ of LL‐37 against *S. aureus*.^[^ ^33^ ^]^ This difference may be attributed to the experimental conditions and different *S. aureus* strains. However, 512 µg mL^−1^ of GMO cubosomes reduced the *S. aureus* population by 1‐log (Figure 3C). This result agrees with previous studies, showing the antimicrobial activity of GMO at high concentrations.^[^ ^34^, ^39^ ^]^ F127 alone at 51.2 µg mL^−1^, as used in the GMO dispersions, did not impact *S. aureus* viability (Figure S17, Supporting Information), confirming GMO's crucial role in the observed antimicrobial activity. Interestingly, combining 512 µg mL^−1^ of LL‐37 and GMO at a 5/5 ratio did not significantly reduce the *S. aureus* population, suggesting an antagonist effect of LL‐37 on GMO's antimicrobial activity. Similar results were obtained with the LL‐37/GMO 2/8 system, in which 2048 µg mL^−1^ of GMO killed *S. aureus* by more than 1‐log, but the addition of 512 µg/mL of LL‐37 inhibited this reduction (Figure S19, Supporting Information).

### 2.4 Impact of LL‐37/GMO Self‐Assemblies Against Biofilms

Confocal microscopy imaging and Live/dead staining were used to confirm the integration of LL‐37/GMO 10/0 and 9/1 elongated micelles into *E. coli* biofilms, which appeared within 30 min of contact (Figure S20, Supporting Information). The antibiofilm activity of the LL‐37/GMO self‐assemblies was then assessed against *P. aeruginosa* and *S. aureus* biofilms. The biofilms were grown for 24 h, followed by 24 h of treatment with different LL‐37/GMO particles. Unformulated LL‐37 exhibited very low biofilm removal activity against *P. aeruginosa*, with ≈20% reduction of the biofilm biomass with 256 µg mL^−1^ using the CV method (Figure S21, Supporting Information). The LL‐37/GMO micelles at 9/1 and 8/2 ratios exhibited a similar biofilm reduction to the unformulated LL‐37, but increasing the GMO content diminished the LL‐37 antibiofilm activity. The vesicles at LL‐37/GMO 5/5 ratio led to ≈6% biofilm reduction. Conversely, 256 µg mL^−1^ of GMO cubosomes slightly promoted *P. aeruginosa* biofilm re‐growth (Figure S21, Supporting Information). The stabilizer F127, when tested in its free form rather than bound to the complex, demonstrated a minimal impact on *P. aeruginosa* biofilms at equivalent concentrations to those used in the LL‐37/GMO systems (Figure S22, Supporting Information).

**Figure 4:**
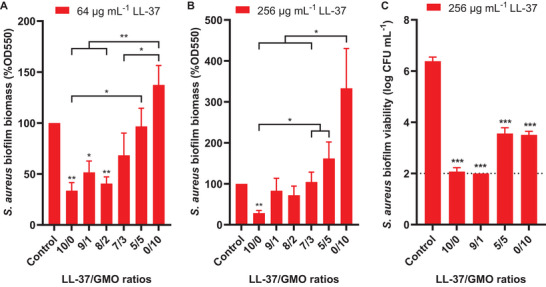
Antibiofilm impact of LL‐37/GMO nanocomplexes against *S. aureus* established biofilms according to the drug‐to‐lipid ratio. 24h‐old biofilms were treated withPBS solutions supplemented with various LL‐37/GMO ratios with concentrations of LL‐37 fixed at A) 64 and B/C) 256 µg mL^−1^ and stabilized with 10% wt F127 relative to LL‐37. “0/10” represents GMO at equivalent concentrations to LL‐37 stabilized with F127. (A/B) After 24 h treatment, the remaining biofilm biomass was measured by CV staining and normalized to the biomass of the control set as 100%. C) After 24 h treatment, biofilm viability was measured by extracting biofilm cells through sonication and CFU counting. The results represent the mean ± SD of (AB) three independent biological repeats for CV staining, and (C) two biological repeats with two readouts each for biofilm viability. Student *t*‐tests were performed using the biomass of the untreated control as a reference or between two conditions with ^*^ *P* < 0.05,^**^ *P* < 0.01, and ^***^ *P* < 0.001.

In contrast to *P. aeruginosa*, unformulated LL‐37 reduced *S. aureus* established biofilms by ≈70% with 64 and 256 µg mL^−1^ from the CV method (**Figure** **4**A,B). This agrees with the literature, which shows that LL‐37 can exhibit antibiofilm activity against *S. aureus* at concentrations lower than its MIC.^[^ ^14^ ^]^ Similar reductions were observed with the LL‐37/GMO 9/1 and 8/2 micelles containing 64 µg mL^−1^ of LL‐37.

Complementary antibiofilm assays were performed using sonication and CFU counting. These measurements confirmed a > 4‐log reduction of viable biofilm cells after treatment with 256 µg mL^−1^ of LL‐37 in LL‐37/GMO 10/0 and 9/1 ratios (Figure 4C). However, the systems prepared as vesicles with an LL‐37/GMO 5/5 ratio showed low antibiofilm activity against *S. aureus* (Figure 4A–C). The biofilm biomass was increased, while the number of viable biofilm cells was reduced by 2‐log after treatment with LL‐37/GMO 5/5 and 0/10. This discrepancy is likely attributed to the role of GMO in structuring biofilm matrix as well as the potential interference of GMO on the CV staining (Figure S15, Supporting Information). The GMO cubosomes promoted *S. aureus* biofilm biomass by 233% with 256 µg mL^−1^. It is unclear whether this biomass increase is due to an increased biofilm regrowth or a GMO‐triggered structural change in the biofilm matrix. Equivalent concentrations of F127 in its free form increased *S. aureus* biofilms by ≈30%, a less pronounced effect than the GMO cubosomes (Figure S22, Supporting Information). These findings demonstrate the critical role of the GMO content in antimicrobial structures, as too high concentrations can inhibit the antibiofilm activity of the LL‐37 and even promote bacterial biofilm mass.

## 3 Discussion

**Figure 5:**
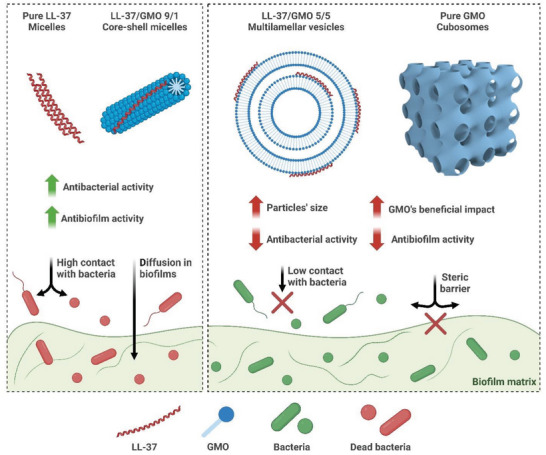
Overview of the biological and physicochemical characteristics of LL‐37/GMO nanocomplexes according to the drug‐to‐lipid ratio. High LL‐37/GMO ratios (e.g., LL‐37/GMO 9/1 and 8/2) led to the formation of small core–shell micelles showing great antimicrobial and antibiofilm activity. Low LL‐37/GMO ratios (e.g., LL‐37/GMO 5/5 and 2/8) led to the formation of vesicles and more complex structures such as cubosomes, gradually inhibiting LL‐37's antimicrobial activity. Schema created in BioRender (BioRender.com/165k585). The schematic illustration of the cubosome is adapted from Freire et al.^[^ ^49^ ^]^ with permission.

AMPs represent a promising alternative to conventional antibiotics, and their efficacy against bacterial infections can be maximized when encapsulated within lipid‐based nanoparticles.^[^ ^6^, ^16^, ^17^, ^33^, ^34^, ^43^ ^]^ This paper investigates how the active peptide‐to‐lipid ratio of LL‐37 and GMO and, ultimately, their structural properties in solution impact their antimicrobial activity. The main findings are summarized in **Figure** **5**.

LL‐37 forms cylindrical micelles (Figure 1). Adding GMO at LL‐37/GMO 9/1, 8/2, and 7/3 ratios leads to core–shell‐type cylindrical micelles with growing dimensions. Above LL‐37/GMO 7/3, multilamellar structures (vesicles), with dimensions >50 nm from DLS analysis, start to coexist with the smaller micelles. The appearance of multilamellar vesicles and micelles at LL‐37/GMO 5/5 agrees with previous findings.^[^ ^30^ ^]^ Further increase in GMO leads to sponge‐like particles and cubosomes.^[^ ^26^, ^28^, ^29^, ^34^ ^]^

The biological assays show a significantly reduced antimicrobial activity against *P. aeruginosa* planktonic cells upon increasing the GMO content at constant LL‐37 concentration (Figure 3). A similar trend was observed with biofilms, with reduced ability of the GMO‐rich vesicles to eradicate *S. aureus* biofilms (Figure 4). The cylindrical micelles obtained with LL‐37/GMO 10/0, 9/1, and 8/2 ratios showed the highest killing activity. In these structures, LL‐37 is located on the surface of the micelles and can directly interact with the bacteria membrane. However, it is also prone to enzymatic degradation in this system (Figure S14, Supporting Information). The finding on reduced antimicrobial activity of LL‐37/GMO self‐assemblies in the form of cubosomes was reported in previous studies (e.g., inactivity of LL‐37/GMO 5/95 cubosomes reported against *E. coli* planktonic cells).^[^ ^26^, ^27^, ^34^ ^]^ Overall, these results suggest that the vesicles reduce the LL‐37 activity, likely due to decreased uptake dynamic and contact surface area with bacteria. Smaller cylindrical micelles diffuse much faster and shuttle the LL‐37 more efficiently to the bacterial membrane. Previous reports have demonstrated an enhanced killing activity of LL‐37/GMO 5/5 systems compared to LL‐37 alone against *E. coli* planktonic cells.^[^ ^26^, ^33^ ^]^ This was not observed in this study, potentially due to the difference in bacterial strains and treatment duration (less than 6 h versus 24 h in this study). In summary, an excess of GMO relative to LL‐37 content must be avoided to maximize the nanocomplex's antimicrobial activity, as demonstrated in this study with LL‐37/GMO ratios of 8/2 and below.

The ζ‐potential of the nanocomplexes is ≈+ 17 mV across all tested ratios (LL‐37/GMO 9/1 to 5/5). This may result from their composition‐dependent self‐assembly into the different structures (**Table** **2**). Generally, cationic liposomes demonstrate better interaction with bacteria and higher antimicrobial activity than uncharged liposomes.^[^ ^21^, ^44^ ^]^ Furthermore, positively charged particles usually show slow penetration within the biofilm matrix due to their immobilization on extracellular DNA.^[^ ^13^, ^45^ ^]^ Despite these characteristics, the antibacterial and antibiofilm activity of the different LL‐37/GMO systems was uncorrelated to their ζ‐potential. This observation suggests that the structure of the antimicrobial nanomaterials triggers the modifications in their antimicrobial activity.

Along with structural properties, the impact of GMO on bacterial physiology can also modulate the activity of the nanoparticles. GMO cubosomes showed contrasting effects on *P. aeruginosa* and *S. aureus*. Elevated concentrations of GMO enhanced both planktonic growth and biofilm formation of *P. aeruginosa* (Figure 2; Figure S21, Supporting Information), indicating a direct counter‐effect to the LL‐37 killing activity. This agrees with previous reports demonstrating that bacteria can use exogenous lipids as energy or carbon sources, notably through the glyoxylate bypass metabolic pathway.^[^ ^46^, ^47^ ^]^ Moreover, exposing *P. aeruginosa* to exogenous polyunsaturated fatty acids has been shown to increase growth and alter its phospholipid profile.^[^ ^48^ ^]^ Consequently, GMO can be counterproductive in antimicrobial nanocarriers and its exact role in bacterial growth remains to be fully elucidated. On the other hand, GMO cubosomes exhibited a lethal impact against *S. aureus* planktonic cells (Figures 2, 3; Figure S19, Supporting Information). This agrees with previous reports demonstrating GMO's antimicrobial impact against *S. aureus in vitro* (2‐log reduction of a planktonic population with ≈1 mg mL^−1^ of GMO)^[^ ^39^ ^]^ and in an *ex vivo* wound infection model.^[^ ^34^ ^]^ The mechanism by which GMO kills *S. aureus* cells is unknown but is likely due to the amphiphilic nature of GMO compromising the bacterial membrane stability. Interestingly, the association of LL‐37 and GMO (at all tested ratios) reduced GMO antibacterial activity against *S. aureus* (Figure 3; Figure S19, Supporting Information), suggesting that the activity is dependent on the nanostructure. The different effects of GMO on both tested bacteria can be attributed to a different metabolism and membrane structure. In contrast, the impact of GMO cubosomes on *S. aureus* biofilms was more complex, inhibiting *S. aureus* biofilm formation (Figure 2) but increasing the biomass of established biofilms (Figure 4). This may be attributed to the killing of dispersed cells blocking bacterial aggregation during biofilm formation and its incorporation in the biofilm network without adversely affecting biofilm cells. The difference in media (i.e., MHB for biofilm formation and PBS for biofilm treatment) could also affect the observed impact. In summary, GMO cubosomes can be strongly beneficial for bacteria, not only impacting the LL‐37 interactions with bacteria but also promoting bacterial growth and biofilm structure.

## 4 Conclusion

The colloidal properties of LL‐37/GMO self‐assemblies are critical determinants in their antimicrobial and antibiofilm activity. Increasing the GMO content relative to constant LL‐37 concentrations by mixing cubosomes and LL‐37 led to structural transformations of the self‐assemblies, ranging from small rod‐like micelles at high LL‐37 content to large multilamellar vesicles around LL‐37/GMO of 5/5. This transition significantly reduced the antimicrobial and antibiofilm activity. The antimicrobial activity was compromised when the GMO content was higher than LL‐37. The results suggest that the colloidal structures are primarily responsible for altering the antimicrobial properties of the self‐assemblies, hindering LL‐37 interaction with bacteria membranes. GMO‐rich vesicles with diameters above 50 nm exhibited reduced antibiofilm activity compared to small micelles with dimensions ≈10 nm, likely due to compromised penetration within the biofilm matrix. Furthermore, our findings demonstrate that a high GMO content can be beneficial for bacterial physiology, promoting *P. aeruginosa* growth, potentially as a nutrient source, and contributing to *S. aureus* biofilm structuring. This study offers novel insights into using lipid‐based self‐assemblies in biofilm treatment, emphasizing that inadequate preparation can unintentionally protect biofilm cells by impairing drug diffusion. Overall, these results highlight the intricate interplay between composition, structure, and function in LL‐37/GMO nanomaterials, offering a novel perspective in the design of lipid‐based antimicrobial therapeutics. Future work could explore fine‐tuning LL‐37/GMO ratios to maintain protection against peptide degradation while maintaining optimal antibacterial activity and drug penetration within the biofilm matrix. This approach can potentially pioneer novel nanomaterials for efficient drug delivery and antimicrobial strategies.

## 5 Experimental Section

### Bacteria and Chemicals

*P. aeruginosa* (MPAO1, Manoil Lab),^[^ ^40^ ^]^ *S. aureus* (ATCC 35556),^[^ ^42^ ^]^ and *E. coli* (DSM 5210) were routinely grown in Mueller Hinton Broth (MHB, 70192, Sigma–Aldrich, Spain) at 37 °C. *P. aeruginosa* biofilms were grown in M9 medium (M9 minimal salts 5×, M6030, Sigma–Aldrich, USA) containing 48 mm Na_2_HPO_4_, 22 mM KH_2_PO_4_, 9 mM NaCl, 19 mM NH_4_Cl, and supplemented with 2 mM MgSO_4_ (63140, Sigma–Aldrich, Germany) 100 µM CaCl_2_ (102382, Millipore, Germany) and 20 mM glucose (G7528, Sigma–Aldrich, USA).^[^ ^41^ ^]^ *S. aureus* biofilms were grown in 10% Tryptic Soy Broth (TSB, 211825, BD Bacto, France) and 0.1% glucose.^[^ ^50^ ^]^ LL‐37 was purchased from Caslo Aps, Denmark (95% purity). Glyceryl monooleate (GMO, C_21_H_40_O_4_, molecular weight = 356.5 g mol^−1^) was supplied by DANISCO, Denmark. Pluronic F127 was obtained from Sigma, St Louis, MO, U.S.A. Phosphate buffered saline (0.02 M, pH 7.4) contained 2.74 mM NaCl (Acros Organics, 99.5% purity, Denmark), 0.054 mm KCl (Carl Roth, Karlsruhe, Germany), 0.2 mM Na_2_HPO_4_.2H_2_O (Sigma–Aldrich, Steinheim, Germany), and 0.036 mM KH_2_PO_4_ (Sigma–Aldrich, Steinheim, Germany). NaOH pellets (>99% purity) were procured from Sigma–Aldrich and a ≈37% HCl stock solution (analytical reagent grade) was obtained from Fischer Scientific, U.S.A. The pH of the buffer and the samples were adjusted using 1 M NaOH and 1 M HCl solutions. Ultra‐pure water (resistivity > 18MΩcm) from Sartorius arium Mini Lab Water Systems (Göttingen, Germany) was used for the preparation of the buffer. All chemicals were used without further purification.

### Sample Preparation

GMO cubosomes were prepared at 80 mg mL^−1^ in PBS buffer at pH 7.0 with 8 mg mL^−1^ of Pluronic F127 (10% wt F127 relative to GMO). GMO was melted at 50 °C within a few minutes and mixed with buffer containing the F127. F127‐stabilized GMO suspensions were homogenized using ultrasonication with a tip sonicator (Lab500 NexTgen Ultrasonic platform, SinapTec, Lezennes, France) at 20% amplitude for 2 min in pulse mode (3 s pulse, 3 s break). LL‐37 stock suspensions were prepared at 50 mg mL^−1^ in PBS buffer at pH 7.0 by vortex mixing. F127 stock suspensions were prepared at 8 mg mL^−1^ in PBS by vortex mixing and were added in the final LL‐37/GMO preparations to keep F127 concentration constant (i.e., 10% F127 relative to LL‐37). The different LL‐37/GMO ratios (9/1, 8/2, 7/3, 5/5, 2/8) were prepared by mixing the LL‐37, F127, and cubosomes in PBS at room temperature. The pH of all preparations was adjusted to pH 7.0 before use. F127‐free cubosome stock suspensions were prepared as previously reported.^[^ ^28^ ^]^ Cubosomes were prepared by dispersing GMO in ultrapure water at 10 mg mL^−1^ followed by consecutive ultrasonication. Three cycles of 5 min ultrasonication in pulse mode (3 sec pulse, 2 sec break) were performed at 7, 7, and 15% amplitude to obtain stable dispersions.

### Planktonic Cells Killing Assay

The antimicrobial activity of LL‐37/GMO self‐assemblies was assessed against *P. aeruginosa* and *S. aureus* following the Clinical and Laboratory Standards Institute (CLSI) guidelines and the broth microdilution protocol of Wiegand et al.^[^ ^51^, ^52^ ^]^ LL‐37 and GMO dispersions were UV‐sterilized for 20 min before use and diluted in PBS at pH 7.0 at twice the desired concentration. Bacteria were prepared from an overnight culture in MHB and diluted in MHB 2X at 1 × 10^6^ CFU mL^−1^. 50 µL of both bacteria and LL‐37/GMO dispersions were mixed in 96 well plates (TPP tissue culture 96 well plates, Z707902, Sigma–Aldrich), resulting in a final concentration of 5 × 10^5^ CFU mL^−1^, LL‐37/GMO 1X, and MHB 1X. The microtiter plates were incubated for 24 h at 37 °C without shaking. The number of living bacteria after treatment was quantified by CFU counting.

### Biofilm Removal Assay

The antibiofilm activity of LL‐37/GMO self‐assemblies was assessed against biofilms formed by *P. aeruginosa* and *S. aureus*, as previously described.^[^ ^41^, ^50^, ^53^ ^]^ Biofilms were grown by incubating 100 µL of bacterial suspensions at 1 × 10^6^ CFU mL^−1^ in 96 well plates for 24 h at 37 °C without shaking. *P. aeruginosa* and *S. aureus* biofilms were grown in an M9 medium^[^ ^41^ ^]^ and 10% TSB / 0.1% glucose, respectively.^[^ ^50^ ^]^ Following 24 h growth, biofilms were washed once with 100 µL of 0,9% NaCl. Washed biofilms were treated with 100 µL of PBS supplemented with various LL‐37/GMO ratios and increasing concentrations of LL‐37 at pH 7.0. After 24 h treatment at 37 °C, the biofilm biomass on the walls of the microtiter plates was washed once with NaCl and stained by crystal violet (0.1%, 20 min, V5265, Sigma–Aldrich). The stained biofilm biomass was washed three times with NaCl to remove unbound CV. The remaining CV dye was dissolved in 70% ethanol and quantified by optical density at 550 nm using a spectrophotometer (Spark 10M, Tecan, Austria). The quantification of biofilm removal was done using the following formula:

(1) $$\mathrm{Biofilm}\;\mathrm{removal}\;\left(\%\right)=\frac{\mathrm{Tested}\;\mathrm{condition}\;\mathrm{OD}550\times 100}{\mathrm{Control}\;\mathrm{OD}550}$$

To measure biofilm viability post‐treatment, biofilm cells were extracted by sonication as previously published.^[^ ^50^ ^]^ Following the previously described biofilm treatment, biofilms were washed thrice with PBS and the biofilm cells were extracted by sonication for 20 s using Vibra‐CellTM 500 W 20 kHz (Sonics and Materials, Inc., Newtown CT, USA) with a 3 mm microtip at 20% amplitude (1 s pulse, 1 s pause) in 200 µL of PBS. The dispersed bacterial cells were then quantified by CFU count.

### Biofilm Imaging by Confocal Microscopy

Due to safety regulations, the biosafety level 1 strain *E. coli* (DSM 5210) was used. *E. coli* biofilms were grown with a starting inoculum of 5 × 10^5^ CFU mL^−1^ for 72 h in MHB media with media renewal every 24 h in 96 well plates (TPP tissue culture 96 well plates, Z707902, Sigma–Aldrich). Biofilms were then rinsed with PBS and treated with LL‐37/GMO self‐assemblies with a fixed LL‐37 concentration of 32 µg mL^−1^ for 30 min. Treated biofilms were then rinsed thrice with PBS and stained with 1 µg mL^−1^ of DAPI (D1306, Invitrogen) and 2 µg mL^−1^ of propidium iodide (537059, Sigma–Aldrich) for 30 min. Stained biofilms were washed to remove unbound dyes and placed in cell imaging dishes (0030 740.017, Eppendorf). Samples were imaged using an inverted spinning disk confocal microscope (Visitron Visiscope CSU‐W1) and a 40x objective (Nikon's CFI series, water immersion, excitation wavelengths of 405 and 515 nm). The images were analyzed with ImageJ.

### Protection Against Enzymatic Degradation

The antimicrobial activity of LL‐37/GMO self‐assemblies was assessed after enzymatic degradation by Proteinase K (740506.75, Macherey‐Nagel) following a published protocol.^[^ ^54^ ^]^ *E. coli* suspensions were prepared from an overnight culture in MHB and diluted in MHB 2X at 1 × 10^6^ CFU mL^−1^. Self‐assemblies were prepared at LL‐37/GMO 10/0, 9/1, and 5/5 ratios with a fixed LL‐37 concentration of 512 µg mL^−1^ and were treated with 20 µg mL^−1^ of proteinase K for 30 min at room temperature. 50 µL of *E. coli* and LL‐37/GMO suspensions post‐treatment were mixed in 96 well plates, resulting in a final concentration of 5 × 10^5^ CFU mL^−1^, 128 µg mL^−1^ of LL‐37 across all systems, and MHB 1X. The microtiter plates were incubated for 24 h at 37 °C without shaking. The number of living bacteria after treatment was quantified by CFU counting.

### ζ‐potential

ζ‐potential measurements were conducted with a Litesizer™ 500 particle analyzer (Anton Paar GmbH, Graz, Austria), employing continuously monitored Phase Analysis Light Scattering (cmPALS). Electrophoretic mobility was determined, and the ζ‐potential was calculated via smoluchowski's theory (Equation 2):

(2) $$\mu_{e}=\frac{\varepsilon_{r}\varepsilon_{0}\zeta }{\eta }$$

where µ_*e*_ is the electrophoretic mobility, ɛ_r_ is the dielectric constant of the medium, ɛ_o_ is the permittivity of the vacuum, and *ζ* is the *ζ*‐potential. All the samples were diluted 1:100 in PBS and re‐adjusted at pH 7.0. All measurements were performed at 25 °C.

### Small‐Angle X‐ray Scattering (SAXS)

SAXS patterns were collected on a SAXSpoint 5.0 system (Anton Paar GmbH, Graz, Austria) connected to an X‐ray source (MetalJet liquid gallium anode with point focus, Excillum, Kista, Sweden) operating at 70 kV and 2.5 mA. A focusing multilayer mirror was used to convert the divergent polychromatic X‐ray beam into a monochromatic focused beam (λ = 0.134 nm). The beam cross‐section diameter was 1 mm. The sample‐to‐detector distance of 1.61 m provided a q‐range of 0.03 to 2.4 nm^−1^, where *q* is the length of the scattering vector, defined by

(3) $$q=\frac{4\Pi }{\lambda }\;\sin\left(\frac{\theta }{2}\right)$$

where *λ* is the wavelength, and θ is the scattering angle. The 2D SAXS patterns were acquired using an Eiger2 R 1 M detector (Dectris Ltd, Baden‐Dättwil, Switzerland) with an active area of 77.1 mm × 79.65 mm and a pixel size of 75 µm × 75 µm. Samples were loaded into a borosilicate glass capillary (diameter = 1.5 mm) and placed in line with the beam. Data were collected from six acquisitions (1800 s each) at 25 °C. The background scattering of PBS was measured and subtracted from all samples.

### Analysis of SAXS Data—Model‐Independent SAXS Data Analysis

The SAXS data were analyzed using the generalized indirect Fourier transformation (GIFT) method, facilitating the estimation of pair‐distance distribution function *p(r)*, which offers insights into the size, shape, and morphology of the particles.^[^ ^36^, ^55^ ^]^ The *p(r)* is connected to the intensity I(q) through Equation 4:

(4) $$I\;\left(q\right)=4\pi \int_{0}^{\infty }p\left(r\right)\frac{\sin\left(qr\right)}{qr}dr$$

For particles of arbitrary shape, the *p*(*r*) =  *r* ^2^Δρ^2^(*r*), with Δρ^2^(*r*) signifying the convolution square of the excess electron density concerning the buffer, averaged over all spatial directions. In the case of monodisperse, homogeneous, and spherical particles, the scattering intensity can then be expressed by

(5) $$I\left(q\right)=NS\left(q\right)P\left(q\right)$$

with N being the number of particles and P(q), the form factor describing the intra‐particle structure and interactions. Inter‐particle effects described by the structure factor S(q) can influence the scattering function at higher concentrations (volume fractions). The GIFT method allows the separation of form and structure factors.^[^ ^55^, ^56^ ^]^ This technique uses the model‐free approach for the p(r) described above, which corresponds only to the form factor. Simultaneously, a model for the structure factor is fitted to obtain a fit of Equation 5 to the data. The effective inter‐particle structure factor for spherical particles interacting through hard sphere (excluded volume) interactions was calculated using the Percus‐Yevick closure relation.^[^ ^57^ ^]^

To calculate the Δρ(*r*) from *p*(*r*), a deconvolution process was employed, with a prior assumption regarding the symmetry of the system (spherical, lamellar, or cylindrical).^[^ ^58^, ^59^ ^]^ In the case of cylindrical micelles their cross‐sectional geometry can be studied using the cross‐section pair‐distance distribution function, *p_c_* (*r*).^[^ ^60^ ^]^ The deconvolution of the *p_c_* (*r*) yields the cross‐sectional contrast profile in electron density Δρ_*c*_. These excess electron density profiles (relative to the PBS) provide insights into the internal structure of the scattering objects. Typically, cylindrical micelles exhibit a core–shell type architecture, comprising a hydrophobic core composed of an alkyl chain of the lipid, along with a hydrophilic shell containing counter ions and bound solvent molecules. Any variation in the excess electron density allows for the direct determination of the core radius and shell thickness from the radial contrast profile.

### Analysis of SAXS Data—Model‐Dependent SAXS Data Analysis

The SAXS data were further analyzed by fitting an analytical model representing the structure of the LL‐37/GMO structures.

In the case of cylinders, the following equation was used to calculate the best possible fit of the model to the experimental data:^[^ ^37^ ^]^

(6) $$I\;\left(q\right)=\mathrm{scale}\int_{0}^{\pi /2}F^{2}\left(q,\alpha \right)\sin\left(\alpha \right)d\alpha$$

where

(7) $$F\;\left(q,\alpha \right)=2\left(\Delta \rho \right)V\frac{\sin\left(\frac{1}{2}qL\cos\alpha \right)}{\frac{1}{2}qL\cos\alpha }\frac{2J_{1}\left(qR\sin\alpha \right)}{qR\sin\alpha }$$

α represents the angle between the cylinder axis and the scattering vector magnitude q, *V*  =  Π*R* ^2^ *L* denotes the volume of the cylinder, L is the length of the cylinder, R accounts for the radius of the cylinder, and Δρ is the difference in electron density between the scatterer and the solvent. *J*_1_ is the first‐order Bessel function.

For randomly oriented particles, the model is corrected by Equation 8:

(8) $$F^{2}\left(q\right)=\int_{0}^{\frac{\pi }{2}}F^{2}\left(q,\alpha \right)\sin\left(\alpha \right)d\alpha$$

Initially, the model considers cylinders at a single orientation angle. When the cylinders are randomly oriented, it becomes necessary to integrate. *F* ^2^(*q*,α)sin(α) over all possible angles (from 0 to 90 ° due to symmetry). Thus, it becomes possible to replace *F* ^2^(*q*,α)sin(α) by *F* ^2^(*q*) in Equation 6.

The scale and ρ_*solvent*_ were kept constant at 1  × 10^−9^ (arb.u.) and 0.5 nm^−2^, respectively. The other parameters (length, radius, and cylinder scattering length density) were optimized to achieve the best possible fit of the model to the experimental data (see Table S2 and Figure S3, Supporting Information).

### Analysis of SAXS Data—Lattice Parameter Calculations

Bragg peaks observed in SAXS curves were correlated with the liquid crystalline structure space groups by the assignment of *hkl* Miller indices.^[^ ^61^ ^]^ For cubosomes, the lattice parameter, *a*_*Im*3*m*_, was obtained from the respective Bragg peak positions denoted as *q_hkl_* by using Equation 9. The average and standard deviation were subsequently determined from the visible peaks.

(9) $$a=\frac{2\pi }{q_{hkl}}\sqrt{h^{2}+k^{2}+l^{2}}$$

For a lamellar structure, the interlamellar distance, d, was calculated using the equation:

(10) $$d=\frac{2\pi h}{q_{h}}$$

where h represents the order of the equidistant Bragg peaks and *q_h_* is the corresponding q value of the *h* ^th^ order Bragg peak.

### Analysis of SAXS Data—Guinier Analysis

Guinier analysis was conducted on the SAXS data at different LL‐37/GMO ratios to acquire additional information on the structures. The Guinier approximation,

(11) $$I\left(q\right)=I\left(0\right)\exp\left(-\frac{q^{2}{R_{g}}^{2}}{3}\right)$$

was used to calculate the radius of gyration, *R_g_*. A fit to a plot of ln I(q) vs *q* ^2^ is used to determine the *R_g_* (from the slope of the fit to the data). The radius of gyration of the corresponding cross‐section, *R_gc_*, was calculated from a cross‐section Guinier plot [lnI(q)q] vs *q* ^2^ and the dimension of the largest semiaxis (c) was estimated using Equation 12:

(12) $${R_{g}}^{2}-{R_{gc}}^{2}=\frac{c^{2}}{5}$$

### Cryogenic Transmission Electron Microscopy (Cryo‐TEM)

Three µL of the LL‐37/GMO self‐assemblies with concentrations of LL‐37 fixed at 10 mg mL^−1^ was applied on a holy carbon‐coated grid (Quantifoil 3.5/1), blotted, and vitrified in liquid ethane (Vitrobot, FEI). Samples were imaged on a Tecnai T20 electron microscope (FEI) operating at 200 keV using a side entry cryo‐stage (Gatan 626). Images were recorded on a slow‐scan CCD under low‐dose conditions. The picture's brightness and contrast were improved equivalently across all samples with ImageJ.

### Multi‐Angle Dynamic Light Scattering (DLS)

Multi‐angle DLS measurements were performed with a light scattering goniometer (CGS‐8F, ALV, Langen, Germany) equipped with a solid‐state laser (Coherent Verdi V5, 532 nm wavelength, max. power of 5 W), a single‐mode fiber detection optics (OZ from GMP, Zürich, Switzerland), and 8 fiber‐optic detectors and ALV 7004 correlators with fast expansion (ALV, Langen, Germany). Scattering angles were measured at intervals between 39 and 124 ° in 17 ° steps. Five accumulations with a measurement time of 240 s per sample were performed at a constant temperature of 25 ^○^ *C*. Samples were diluted at a 1:10 ratio with PBS to avoid multiple scattering. The DLS autocorrelation functions were fitted using 2^nd^‐order cumulant analysis to determine the apparent diffusion coefficient (*D*).^[^ ^62^, ^63^ ^]^ Alternatively, the average decay constant $\left(\bar{\Gamma }\right)$ obtained from the correlation functions was plotted against *q* ^2^ and fitted with a linear curve to determine the apparent translational diffusion (D):

(13) $$\bar{\Gamma }=Dq^{2}+c$$

where

(14) $$q=\frac{4\Pi n}{\lambda }\sin\left(\frac{\theta }{2}\right)$$

c is the intercept. The refractive index (n) used for PBS was 1.33. The hydrodynamic radius (R_H_) was determined from *D* using the Stokes‐Einstein equation:

(15) $$R_{H}=\frac{k_{b}T}{6\pi \eta D}$$

where k_b_ is the Boltzmann constant, T is the absolute temperature (298 K), η is the solvent's viscosity (PBS, 1 mPa s). The PDI of the size distribution is determined from the second cumulant (µ_2_) over all angles (between 39 and 124 ° in 17 ° steps):

(16) $$\mathrm{PDI}=\frac{\mu_{2}}{T^{2}}$$

## Conflict of Interest

The authors declare no conflict of interest.

## Supporting information



Supporting Information

## Data Availability

The data that support the findings of this study are available in the supplementary material of this article.
