# Supplementary material for: Colloidal Structure Dictates Antimicrobial Efficacy in LL‐37 Self‐Assemblies With Glycerol Monooleate
Source: Small. 2024 Oct 15;20(51):2405131. doi: 10.1002/smll.202405131 (PMC11657029; doi:10.1002/smll.202405131)
Supplement: Supplementary file 1 — Supporting Information [file SMLL-20-2405131-s001.docx]

**Supplemental information**

**Colloidal Structure Dictates Antimicrobial Efficacy in LL-37 Self-assemblies with Glycerol Monooleate**

Jules D. P. Valentin^1#^*, Parth Kadakia^#1^, Lucie J. Varidel^1^, Marc C. A. Stuart^2^, Stefan Salentinig^1^*

^1^ Department of Chemistry and National Center of Competence in Research Bio-inspired Materials, University of Fribourg, Chemin du Musée 9, 1700 Fribourg, Switzerland

^2^ Centre for System Chemistry, Stratingh Institute for Chemistry and Groningen Biomolecular Science and Biotechnology Institute, University of Groningen, Nijenborgh 7, 9747AG Groningen, The Netherlands

^#^ Contributed equally

* Corresponding authors:

Jules Valentin, email : [jules.valentin@unifr.ch](mailto:jules.valentin@unifr.ch), Tel: +41 79 860 8183

Stefan Salentinig, email : stefan.salentinig@unifr.ch, Tel : +41 26 300 8794

**Table S1:** Effective structure factor parameters calculated from the SAXS data with the GIFT method. The hard sphere model was used for all systems. These models lead to stable *p*(*r*) functions that give access to the required information on the size and shape of the micelles. As long as the mean deviation reaches a global minimum with a parameter set, the calculation of *p*(*r*) and *P*(*q*) will result in correct curves.^1^

| Sample | Volume fraction | Radius [nm] |
| --- | --- | --- |
| LL-37 | 0.035 ± 0.002 | 3.9 ± 0.2 |
| LL-37+F127 | 0.046 ± 0.001 | 4.2 ± 0.1 |
| LL-37/GMO 9/1 | 0.033 ± 0.002 | 4.5 ± 0.2 |
| LL-37/GMO 8/2 | 0.036 ± 0.005 | 5.4 ± 0.3 |
| LL-37/GMO 7/3 | 0.16 ± 0.07 | 3.2 ± 0.8 |

**Table S2.** Fitting parameters for LL-37 with and without 10% F127 relative to LL-37 were analyzed with an analytical form factor model for homogeneous cylinders (equation 6 mentioned in the method section). Scale and $\rho_{solvent}$ were kept constant at ${1 \times10}^{-9}$ (arb.u.) and 0.5 nm^-2^. The length, radius, and cylinder scattering length density of the cylinder were optimized to achieve the best possible fit of the model to the experimental SAXS data (see Figure S3).

|  | LL-37/F127 | LL-37 |
| --- | --- | --- |
| Scale (arb. u.) | ${1 \times10}^{-9}$ | ${1 \times10}^{-9}$ |
| Solvent scattering length density (nm^-2^) | 0.5 | 0.5 |
| Length (nm) | 5.6 ± 0.02 | 5.3 ± 0.01 |
| Radius (nm) | 1.5 ± 0.01 | 1.6 ± 0.01 |
| Cylinder scattering length density (nm^-2^) | 0.07 ± 0.01 | 0.08 ± 0.01 |

**Table S3.** Summary of radius of gyration ($R_{g}$) for different LL-37/GMO elongated structures, after removal of the structure factor scattering.

| Sample | $R_{g}$ (nm) |
| --- | --- |
| LL-37 | 1.9 |
| LL-37+F127 | 1.8 |
| LL-37/GMO 9/1 | 2.4 |
| LL-37/GMO 8/2 | 3.1 |
| LL-37/GMO 7/3 | 4.7 |

**Figure S1.** (A) Experimental SAXS curves (dots) with error bars and the corresponding fits (red line), calculated with GIFT for LL-37 dispersions with and without F127. (B) The calculated *p(r)* functions of LL-37 with and without F127 corresponding to the fits in **Figure S1A** at pH 7.0.

**Figure S2:** Structure factor curves calculated from the experimental SAXS data using the hard sphere model. The corresponding parameters are summarised in **Table S1.**

**Figure S3.** Experimental SAXS data (black dots) and the corresponding model fits (red line) calculated with equation 6 for elongated (A) LL-37 and (B) LL-37/F127 cylinders. The corresponding parameters are presented in Table S2.

**Figure S4.** Selected cryo-TEM micrographs of the LL-37/GMO self-assemblies. LL-37/GMO self-assemblies were prepared with LL-37 fixed concentrations at 10 mg mL^-1^ in PBS 0.02M at pH 7.0.

**Figure S5.** (A) The calculated *p(r)* function from the GIFT analysis, normalized to 1.0 at its maximum, is presented in for LL-37/GMO 7/3 at pH 7.0. (B) The structure factor curve calculated from the experimental SAXS data using the hard sphere model. The corresponding parameters are summarised in **Table S1.**

**Figure S6.** (A) The calculated cylinder cross-section pair distance distribution function, $p_{c}\left( r \right)$, for LL-37/GMO 9/1 (blue symbols). The calculated fit from deconvolution is shown as a red line. (B) Deconvolution of the $p_{c}\left( r \right)$leads to the excess electron density distribution profile for the cylinder cross section. (C) Experimental SAXS curves (black symbols) and the corresponding fit (red line) calculated with IFT for LL-37/GMO 9/1 cylinder cross-section, $p_{c}\left( r \right)$.

**Figure S7.** SAXS scattering curves of LL-37/GMO 9/1 prepared with and without F127. (A) SAXS curves. (B) p(r) from GIFT analysis. (C) The corresponding S(q) curves calculated from the GIFT analysis using the hard sphere model.

**Figure S8.** Impact of storage on LL-37/GMO self-assemblies particle size. The results represent the SAXS scattering curves of LL-37/GMO 9/1 ratio freshly prepared and after three weeks storage at 4°C.

**Figure S9.** Guinier plots of ln P(q) vs $q^{2}$ to determine the radius of gyration ($R_{g}$). The full lines (red) are the calculated best possible fits to the experimental data in the Guinier region for (A) LL-37 without F127, (B) LL-37 with 10% F127 relative to LL-37, LL-37/GMO (D) 9/1, (E) 8/2, and (F) 7/3 combinations. Refer to **Table S3** for detailed information regarding the $R_{g}$ values at various LL-37/GMO ratios.

**Figure S10.** Modified Guinier plots of ln [P(q)q] vs $q^{2}$ used for the calculation of the radius of gyration of the cylinder cross-section ($R_{gc}$) for (A) LL-37 without F127, (B) LL-37 with 10% F127 relative to LL-37, LL-37/GMO (D) 9/1, (E) 8/2, and (F) 7/3 ratios. The full lines (red) are the calculated fits to the experimental data with the vertical lines (pink) representing the data range used for fitting. The curves indicate elongated structures with the linear region at q$R_{gc}$ < 1.1. **Table 2** provides more details about $R_{gc}$ for different LL-37/GMO ratios.

**Figure S11.** Experimental SAXS curve of LL-37/GMO 5/5 ratio showing Bragg peaks (indexed with *) at q = 1.3 nm^-1^, 2.6 nm^-1^, 3.9 nm^-1^ indicating presence of multilamellar structures, likely in the form of oligo-lamellar vesicles.

**
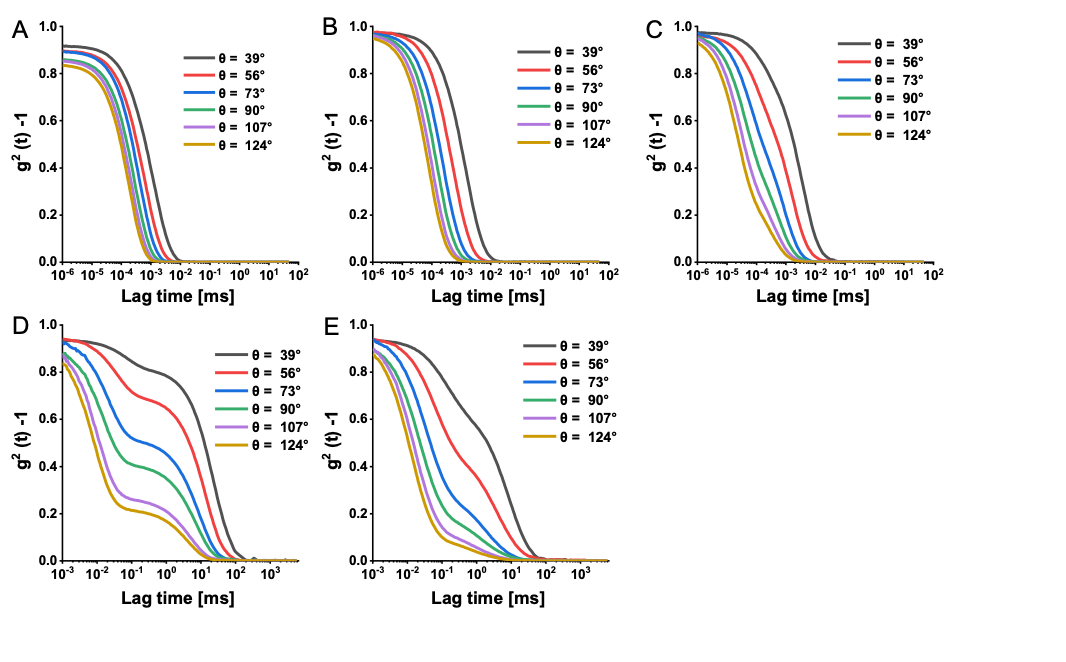
Figure S12.** The DLS intensity correlation functions at scattering angles from 39 to 124° with a delta of 17° measured at 25°C. The curves for (A) GMO and (B) LL-37/GMO 5/5 are dominated by a single decay, indicating mostly monomodal particle size distribution. Multiple decays reflecting multimodal particle size distributions are observed for (C) LL-37/GMO 7/3, (D) LL-37/GMO 9/1 without F127, and (E) LL-37/GMO 9/1 with F127.

**Figure S13.** The decay constant of the DLS autocorrelation function plotted against $q^{2}$ for GMO (black square), LL-37/GMO 7/3 (red circle), and LL-37/GMO 5/5 (blue triangle) with the corresponding linear fits for translational diffusion. The reasonable representation of the experimental data with the linear fit for GMO and LL-37/GMO 5/5 is characteristic of a mostly monomodal size distribution. The deviation of the LL-37/GMO 7/3 indicates multi-modal distributions, in agreement with the discussion of the corresponding autocorrelation functions in Figure S9. The apparent translational diffusion (D) was determined from the slope of the linear fit for each dispersion. The D value was used to further determine the R_H_ using the Stokes-Einstein equation, which is detailed in the Method Section.

**Figure S14.** Antimicrobial activity of LL-37/GMO self-assemblies against *Escherichia coli* planktonic cells after enzymatic degradation. Self-assemblies were prepared at LL-37/GMO 10/0, 9/1, and 5/5 ratios with a fixed LL-37 concentration of 512 µg mL^-1^ and were treated with 20 µg mL^-1^ of proteinase K for 30 minutes at room temperature. The LL-37/GMO suspensions were further diluted down to 128 µg mL^-1^ of LL-37 and immerse in *E. coli* suspensions in Mueller Hinton Broth (MHB). The antimicrobial activity of all systems was then determined by quantifying the number of viable planktonic cells of *E. coli* after 24h treatment at 37°C by colony-forming units (CFU) counting. The results represent one biological repeat, with the dashed line representing the limit of detection of the assay.

**Figure S15.** Impact of GMO pretreatment on crystal violet (CV) staining and biofilm biomass quantification. *P. aeruginosa* and *S. aureus* biofilms were formed in M9 and 10%TSB+0.1%Glucose media for 24h at 37°C, respectively. After 24h, biofilms were rinsed with PBS and immersed in GMO suspensions at 0, 256, and 512 µg mL^-1^ in PBS 0.02M at pH 7.0 for 30 minutes. After pretreatment, biofilms were washed once with PBS, and the biomass was quantified by CV (0,1%, 15 min). Blank conditions correspond to CV staining in sterile wells pretreated similarly to biofilms. The results represent the average ± STDS of three independent biological repeats with two readouts each. Student *t*-tests were performed using the control as a reference with * P < 0.05, ** P < 0.01, and *** P < 0.001.

**Figure S16.** Viability of (A) *P. aeruginosa* and (B) *S. aureus* after 24 h culture in the presence of increasing concentrations of LL-37 and LL-37/F127 (10% F127 relative to LL-37). Bacterial viability was measured by counting the CFU mL^-1^ after 24 h culture in MHB media supplemented with LL-37 or LL-37/F127 at pH 7.0 and 37°C. The results represent the mean ± SD of three independent biological repeats. The dashed line represents the limit of detection of the assay. Student *t*-tests were performed using the viability of the control as a reference or between two conditions with * P < 0.05, and *** P < 0.001.

**Figure S17.** Viability of (A) *P. aeruginosa* and (B) *S. aureus* after 24 h culture in the presence of F127. Bacterial viability was measured by counting the CFU mL^-1^ after 24 h culture in MHB media supplemented with F127 at pH 7.0 and 37°C. The results represent the mean ± standard deviations (SD) of three independent biological repeats. The dashed line represents the limit of detection of the assay.

**Figure S18.** Impact of storage on LL-37/GMO self-assemblies activity. The antibacterial activity of LL-37/GMO self-assemblies was assessed against *P. aeruginosa* planktonic cells with a freshly prepared stock and after 1-week storage at 4°C. The results represent the mean ± standard deviations (SD) of three independent biological repeats. The dashed line represents the limit of detection of the assay. Student *t*-tests were performed using the viability of the control as a reference with * P < 0.05 and *** P < 0.001.

**Figure S19.** Viability of *S. aureus* after 24 h culture in the presence of 2048 μg/mL of GMO associated or not with 512 μg/mL LL-37 (LL-37/GMO 2/8 ratio). Bacterial viability is measured by counting the CFU mL^-1^ after 24 h culture in MHB media supplemented with GMO or LL-37 at pH 7.0 and 37°C. The results represent the mean ± SD of three independent biological repeats. Student *t*-tests were performed using the viability of the control as a reference or between two conditions with ** P < 0.01, and *** P < 0.001.

**Figure S20.** Confocal microscopy pictures of *E. coli* biofilms after treatment with LL-37/GMO self-assemblies. *E. coli* biofilms were grown for 72h in MHB media with media renewal every 24h in 24-well plates. Biofilms were then rinsed with PBS and treated with LL-37/GMO self-assemblies with a fixed LL-37 concentration of 32 µg mL-1 for 30 min. Treated biofilms were then rinsed thrice with PBS and stained with 1 µg mL^-1^ of DAPI (D1306, Invitrogen) and 2 µg mL^-1^ of propidium iodide (537059, Sigma-Aldrich) for 30 min. Stained biofilms were then washed to remove unbound dyes and placed in cell imaging dishes (0030 740.017, Eppendorf). Samples were imaged using an inverted spinning disk confocal microscope (Visitron Visiscope CSU-W1) and a 40x objective (Nikon’s CFI series, water immersion, excitation wavelengths of 405 and 515 nm).). The images were analyzed with ImageJ.

**Figure S21.** Antibiofilm activity of LL-37/GMO self-assemblies against *P. aerug*inosa established biofilms determined by (A) crystal violet staining and (B) colony forming units (CFU) counting. Biofilms were formed during 24h in M9 media in 96-well plates. Biofilms were then treated for 24h with increasing concentrations of LL-37 prepared in various LL-37/GMO ratios in PBS 0.02M at pH 7.0. Following treatment, biofilms were washed thrice with NaCl 0.9% to remove planktonic cells and drugs. (A) The remaining biofilm biomass post-treatment was quantified by CV staining (0,1%, 15 min) and normalized to the biomass of the control set as 100%. The results represent the mean ± SD of three independent biological repeats. (B) Biofilm viability post-treatment was evaluated by extracting biofilm cells by sonication (amplitude 20%, 1 sec pulse 1 sec break over 20 seconds total) and CFU counting. The results represent the mean ± standard deviations (SD) of one independent biological repeat with two readouts. The dashed line represents the limit of detection of the assay. Student *t*-tests were performed using the viability of the control as a reference with * P < 0.05 and ** P < 0.01.

**Figure S22.** Antibiofilm impact of F127 against (A) *P. aeruginosa* and (B) *S. aureus* established biofilms. Biofilms were formed during 24 h, washed with NaCl solution, and treated with phosphate-buffered saline (PBS) supplemented with F127 at pH 7.0 and 37°C. After 24 h treatment, the remaining biofilm biomass was measured by CV staining and normalized to the biomass of the control set as 100%. The results represent the mean ± SD of three independent biological repeats. Student *t*-tests were performed using the biomass of the untreated control as a reference with * P < 0.05.

**References**

1. Fritz, G.; Bergmann, A.; Glatter, O., Evaluation of small-angle scattering data of charged particles using the generalized indirect Fourier transformation technique. *The Journal of Chemical Physics* **2000,** *113* (21), 9733-9740.
